# Disclosing α‐lactalbumin impact on the intestinal and vaginal microbiota of women suffering from polycystic ovary syndrome

**DOI:** 10.1111/1751-7915.14540

**Published:** 2024-10-04

**Authors:** Giulia Alessandri, Leonardo Mancabelli, Federico Fontana, Elisa Lepore, Gianpiero Forte, Moira Burratti, Marco Ventura, Francesca Turroni

**Affiliations:** ^1^ Laboratory of Probiogenomics, Department of Chemistry, Life Sciences, and Environmental Sustainability University of Parma Parma Italy; ^2^ Department of Medicine and Surgery University of Parma Parma Italy; ^3^ Microbiome Research Hub University of Parma Parma Italy; ^4^ R&D Department Lo.Li Pharma Rome Italy; ^5^ A.G.UN.Co. Obstetrics and Gynaecology Center Rome Italy

## Abstract

Polycystic ovary syndrome (PCOS) is one of the most widespread endocrinopathy affecting women of reproductive age with detrimental effects on life quality and health. Among several mechanisms involved in its aetiopathogenesis, recent studies have also postulated the involvement of the vaginal and intestinal microbiota in the development of this disorder. In this study, an accurate insight into the microbial changes associated with PCOS was performed through a pooled‐analysis highlighting that this syndrome is characterized by intestinal and vaginal dysbiosis with a reduction of beneficial microorganisms and a higher proportion of potential pathogens. Based on this observation, we evaluated the ability of a milk‐derived protein exerting positive outcomes in the management of PCOS, that is, α‐lactalbumin (α‐LA), to recover PCOS‐related dysbiosis. In vitro experiments revealed that this protein improved the growth performances of members of two health‐promoting bacterial genera, that is, *Bifidobacterium* and *Lactobacillus*, depleted in both intestinal and vaginal microbiota of PCOS‐affected women. In addition, α‐LA modulated the taxonomic composition and growth performances of the microbial players of the complex intestinal and vaginal microbiota. Finally, an in vivo pilot study further corroborated these observations. The oral administration of α‐LA for 30 days to women with PCOS revealed that this protein may have a role in favouring the growth of health‐promoting bacteria yet limiting the proliferation of potential pathogens. Overall, our results could pave the way to the use of α‐LA as a valid compound with ‘prebiotic effects’ to limit/restore the PCOS‐related intestinal and vaginal dysbiosis.

## Graphical abstract

The milk‐derived protein α‐lactalbumin exerts beneficial effects in women suffering from PCOS by improving the growth performances of certain health‐promoting bacteria and modulating the taxonomic composition of both intestinal and vaginal microbiota.

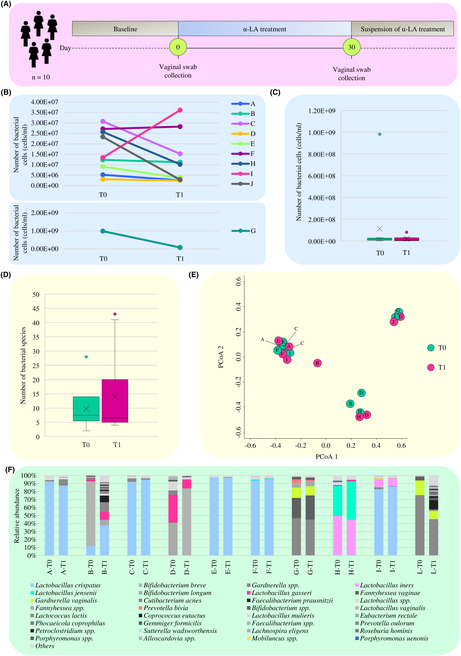

## INTRODUCTION

Polycystic ovary syndrome (PCOS) is one of the most common chronic endocrinopathies affecting women of reproductive age worldwide (Mukherjee et al., 2023; Sadeghi et al., 2022; Zhu & Goodarzi, 2022). Besides hyperandrogenism, polycystic ovaries and/or ovulatory dysfunction, a plethora of comorbidities may accompany such syndrome, including hirsutism, acne, cardiovascular problems, high incidence rate of breast and endometrial cancer, high risk for infertility and pregnancy complications, as well as various metabolic alterations such as obesity, insulin resistance, hyperinsulinaemia and type 2 diabetes mellitus (Barthelmess & Naz, 2014; Cardinale et al., 2022; Gu et al., 2022; Parker et al., 2022b; Rajska et al., 2020). However, despite the long‐term and disabling repercussions on both health and life quality of PCOS‐affected women, the mechanisms regulating the occurrence of this syndrome are still far from being fully understood (Thackray, 2019; Yurtdas & Akdevelioglu, 2020). In this context, while environmental, epigenetic and hereditary influences are widely investigated (De Leo et al., 2016; Escobar‐Morreale, 2018; Patel, 2018; Siddiqui et al., 2022), emerging evidence highlighted the critical role of the gut microbiota in the pathogenesis of this syndrome (Cardinale et al., 2022; Parker et al., 2022a; Yurtdas & Akdevelioglu, 2020). Indeed, several studies revelled a clear and strong association between the dysbiosis of gut microbiota, that is, the microbial ecosystem inhabiting the human intestine, and multiple alterations in clinical‐metabolic parameters in women affected by PCOS, leading to the formulation of the PCOS microbiological theory, also known as ‘dysbiosis of gut microbiota’ (DOGMA theory) (Gu et al., 2022; Insenser et al., 2018; Torres et al., 2018; Tremellen & Pearce, 2012). According to the latter, the disruption of the gut microbiota eubiosis favours an increased gut mucosal permeability with a parallel increment of lipopolysaccharide release, produced by intestinal Gram‐negative bacteria into the systemic circulation, leading to the activation of the host immune system that responds soliciting an inflammatory status responsible for the onset of various comorbidities (Insenser et al., 2018; Qi et al., 2021; Sanchez‐Garrido & Tena‐Sempere, 2020; Zhao et al., 2020). More recently, a clinical study also demonstrated that regardless of PCOS phenotype, if hyperandrogenic or not (Myers et al., 2023), gut microbiota results to be altered when compared to healthy individuals (Suturina et al., 2022).

In addition, recent studies demonstrated that also vaginal microbiota undergoes important alterations in PCOS‐affected women with a significant reduction of the genus *Lactobacillus*, generally associated with a healthy status, and a parallel increment of potential pathogens such as *Gardnerella vaginalis* and the genera *Prevotella* and *Mycoplasma* (Gu et al., 2022; Lu et al., 2021; Mukherjee et al., 2023; Tu et al., 2020; Wang et al., 2021). However, despite growing scientific evidence suggesting the association between PCOS and altered vaginal and intestinal microbial ecosystems, there are still few studies concerning the characterization of the intestinal and vaginal microbiota in such patients.

Among the natural molecules with positive impact on the management of PCOS, the scientific community has recently become interested in α‐lactalbumin (α‐LA) (Cardinale et al., 2022; Tinghall Nilsson et al., 2024; Zapata et al., 2017) not only for its physical characteristics of water solubility and heat stability, which make it an easy‐to‐use ingredient for the formulation of food supplements, but also for its low immunogenicity, high nutritional value and ability to stimulate health‐promoting effects. Indeed, α‐LA is a human milk‐derived protein that reaches unchanged the intestine where it can be subjected to proteolytic digestion by pancreatic enzymes, including pepsin, trypsin and chymotrypsin, releasing not only bioactive peptides with antibacterial, anti‐inflammatory, analgesic and immunomodulatory effects, but also its peculiar aminoacidic building‐block, encompassing tryptophan, lysine, cysteine and branched‐chain amino acids (Kamau et al., 2010; Krissansen, 2007; Layman et al., 2018; Pellegrini et al., 1999; Yamaguchi et al., 2009). Furthermore, thanks to its positive effect on the permeability of intestinal tight junctions, α‐LA improves the intestinal absorption of some micronutrients, including inositols, which are natural molecules generally used in combination with α‐LA to treat PCOS, consequently improving clinical outcomes in PCOS‐affected patients (Bizzarri et al., 2022; Cardinale et al., 2022; Kamenov et al., 2023; Monastra et al., 2018; Montanino Oliva et al., 2018). In addition, some recent works suggested the positive role of α‐LA in attenuating the microbial dysbiosis associated with PCOS, inducing an increment in the relative abundance of certain beneficial microorganisms such as *Bifidobacterium* and *Lactobacillus* strains (Boscaini et al., 2019; Chen et al., 2022). However, despite this evidence, the possible impact of the administration of α‐LA on the intestinal and vaginal bacterial communities of women with PCOS, is still far from being fully dissected.

In this context, a pooled‐analysis was first set up to provide an accurate overview of the microbial alterations characterizing the intestinal and vaginal microbiota of women with PCOS. Subsequently, as PCOS condition exhibited a reduced relative abundance of two health‐promoting microbial genera, that is, *Bifidobacterium* and *Lactobacillus*, in the intestinal and vaginal microbial ecosystem, respectively, we investigated, through in vitro experiments, the ability of α‐LA to positively influence the growth performances of members of these two bacterial taxa and to modulate the taxonomic composition and growth performances of the microbial players of the female intestinal and vaginal microbiota. Finally, with the aim to deeply investigate and corroborate beneficial effects of α‐LA, an in vivo pilot study based on α‐LA oral administration to women with clinical signs of PCOS evaluated the ability of this protein to modulate the dysbiotic vaginal microbiota typical of a PCOS condition, thus opening towards new perspective applications of such molecule.

## EXPERIMENTAL PROCEDURES

### Selection of public data sets

To perform a pooled‐analysis aimed at defining possible differences in the taxonomic composition of both faecal and vaginal microbiota between healthy and PCOS‐affected women, an in‐depth literature search was carried out to retrieve all microbiome data sets based on Illumina sequencing technology corresponding to faecal and vaginal samples from healthy and PCOS‐affected women. In case of studies involving the administration of drugs, prebiotics and/or probiotics or hormone treatment, only faecal and vaginal samples belonging to the control group were selected. Furthermore, faecal and vaginal samples belonging to women with pathologies other than PCOS were excluded from the analysis to avoid biases related to these additional pathologies. Based on these parameters, a total of 391 faecal samples, divided into 216 and 175 stool from healthy and PCOS‐affected women, respectively, were selected, together with a total of 274 vaginal swabs, subdivided into 140 and 134 vaginal samples from healthy and PCOS‐affected women, respectively (Tables 1 and S1).

**TABLE 1:** Metadata associated with the faecal and vaginal samples included in the pooled‐analysis.

Study	Bioproject	Number of samples	Type of sample	Average age (days)	Nation	Amplified 16S rRNA variable regions
Ling et al. (2023)	PRJNA904086	13 H	V	40.33 ± 11.60	China	V3‐V4
Kim et al. (2021)	PRJNA745060	32 H	V	39.4 ± 3.2	South Korea	V4
Zhou et al. (2019)	PRJNA548879	20 H	V	34.3 ± 4.1	China	V3‐V4
Ahannach et al. (2021)	PRJEB45093	22 H	V	27	Belgium	V4
Tu et al. (2020)	OEP000469	51 P – 47 H	V	30 ± 4	China	V3‐V4
Hong et al. (2021)	PRJNA699990	89 P	V	26.75	China	V3‐V4
Liu et al. (2017)	SRP085887	34 P – 15 H	F	25.5 ± 4.3 P – 32.2 ± 5.9 H	China	V3‐V4
Suturina et al. (2022)	PRJNA899143	68 P – 131 H	F	29.48 ± 5.21 P – 35.11 ± 5.71 H	Russia	V1‐V3
Lindheim et al. (2017)	SRP077213	24 P – 19 H	F	27 P – 32 H	Austria	V1‐V2
Yu et al. (2022)	PRJNA779930	20 P – 20 H	F	28.95 ± 5.83 P – 26.75 ± 5.46 H	China	V3‐V4
Garcia‐Beltran et al. (2021)	PRJNA659664	29 P – 31 H	F	31	Spain	V3‐V4

Abbreviations: F, faecal sample; H, healthy; P, PCOS; V, vaginal swab.

### 16S rRNA microbial profiling‐based microbiome data set analysis

To avoid biases caused by different bioinformatic analysis pipelines, the sequence read pools of each data set were filtered and re‐analysed through the QIIME2 software (Bokulich et al., 2018; Caporaso et al., 2010). Paired‐end reads were merged, and quality control retained sequences with a length between 140 and 400 bp and mean sequence quality score of >20, while sequences with homopolymers of >7 bp and mismatched primers were omitted. 16S rRNA amplicon sequence variants (ASVs) were defined at 100% sequence homology using DADA2 (Callahan et al., 2016). ASVs that were represented only by a single sequence were removed and all reads were classified to the lowest possible taxonomic rank using QIIME2 (Bokulich et al., 2018; Caporaso et al., 2010) and a reference database from the SILVA database v.132 (Quast et al., 2013). Furthermore, α‐diversity analyses were assessed through the observed ASVs index, while biodiversity analyses were calculated through the Bray–Curtis dissimilarity index and represented through a three‐dimensional principal coordinate analysis (PCoA).

### *Bifidobacterium* and *Lactobacillus* strain growth conditions

*Bifidobacterium* and *Lactobacillus* strains were grown overnight in the De Man‐Rogosa‐Sharpe (MRS) broth (Sharlau Chemie, Spain) supplemented with 0.05% (wt/vol) L‐cysteine hydrochloride at 37°C in an anaerobic chamber (Concept 400; Ruskinn) (2.99% H_2_, 17.01% CO_2_ and 80% N_2_). In detail, 12 *Lactobacillus* and 18 *Bifidobacterium* strains were selected to include only strains isolated from human vaginal and faecal microbiota, respectively, from our microbial repository as well as from international microbial culture collections, encompassing one outlier per considered bacterial genus, that is, *Lactobacillus johnsonii* DSM 20533 and *Bifidobacterium asteroides* LMG 10735, isolated from sour milk and honeybee gut, respectively (Table 2).

**TABLE 2:** List of *Bifidobacterium* and *Lactobacillus* strains grown on α‐lactalbumin.

Strains	Ecological origin
*Lactobacillus johnsonii* DSM 20533	International culture collection (swine waste)
*Lactobacillus crispatus* PRL2021	Vaginal tract
*Lactobacillus crispatus* LB56	Vaginal tract
*Lactobacillus crispatus* LB57	Vaginal tract
*Lactobacillus crispatus* LB61	Vaginal tract
*Lactobacillus jensenii* GL‐2C	Vaginal tract
*Lactobacillus jensenii* V7‐9H	Vaginal tract
*Lactobacillus jensenii* V9‐4G	Vaginal tract
*Lactobacillus gasseri* ATCC9857	International culture collection (vaginal tract)
*Lactobacillus gasseri* GA‐2G	Vaginal tract
*Lactobacillus gasseri* V10‐5C	Vaginal tract
*Lactobacillus iners* LMG 14328	Vaginal tract
*Bifidobacterium asteroides* LMG 10735	International culture collection (honeybee hindgut)
*Bifidobacterium adolescentis* ATCC 15703	International culture collection (adult intestine)
*Bifidobacterium adolescentis* 703B	Faecal sample
*Bifidobacterium adolescentis* 713B	Faecal sample
*Bifidobacterium bifidum* LMG 11041	International culture collection (breast‐fed infant faeces)
*Bifidobacterium bifidum* 324B	Faecal sample
*Bifidobacterium bifidum* PRL2010	Faecal sample
*Bifidobacterium breve* LMG 13208	International culture collection (infant intestine)
*Bifidobacterium breve* 689B	Faecal sample
*Bifidobacterium breve* 1895B	Faecal sample
*Bifidobacterium catenulatum* LMG 11043	International culture collection (sewage)
*Bifidobacterium catenulatum* 1899B	Faecal sample
*Bifidobacterium longum* LMG 13197	International culture collection (adult intestine)
*Bifidobacterium longum* 39B	Faecal sample
*Bifidobacterium longum* 67B	Faecal sample
*Bifidobacterium pseudocatenulatum* LMG 10505	International culture collection (infant intestine)
*Bifidobacterium pseudocatenulatum* 289B	Faecal sample
*Bifidobacterium pseudocatenulatum* 318B	Faecal sample

### *Bifidobacterium* and *Lactobacillus* strain growth assay on α‐lactalbumin

To test the ability of the selected strains to grow on α‐LA (provided by LoLi pharma s.r.l., Rome, Italy), *Bifidobacterium* and *Lactobacillus* strains were inoculated in MRS supplemented with 3%, 2% and 1% (wt/vol) α‐LA in a 96‐well microtitre plate to reach a final optical density at 600 nm (OD_600nm_) of 0.1. The same strains were also inoculated in MRS broth without the addition of α‐LA or any other additional proteins as a control sample. Plates were incubated at 37°C under anaerobic conditions. After 48 h of incubation, cell growth was evaluated monitoring the OD_600nm_ by using a plate reader (Biotek, USA), as previously described (Alessandri et al., 2022b; Lugli et al., 2020; Tarracchini et al., 2021). Briefly, plates were read in discontinuous mode with absorbance readings performed at 3 min intervals three times after 48 h of growth, and each reading was ahead of 30 s of shaking at medium speed. Cultures were grown in triplicates, and the resulting growth data sets were expressed as the average of these replicates. MRS was filter‐sterilized by using a filter with pore size of 0.2 μm when supplemented with α‐LA.

### Faecal and vaginal sample collection

To test the impact of an in vitro treatment with α‐LA on complex bacterial communities, both faecal and vaginal samples were collected from 10 healthy women who had not taken prebiotics, probiotics or drugs during the 3 months prior to sample collection. Approximately 3 g of fresh stool were collected from each woman immediately after defecation using a dedicated sterile tube provided of a sampling spoon and containing 15 mL of sterile PBS (phosphate‐buffered solution, pH 6.5). In parallel, vaginal samples were collected by using a vaginal swab, then preserved in a dedicated sterile tube with 3 mL PBS. In all cases, PBS was pre‐reduced with 0.1% (w/v) L‐cysteine hydrochloride. After collection, samples were immediately shipped to the laboratory under anaerobic conditions and processed within 3 h of collection.

### Gut and vaginal microbiota culturing

Faecal samples were inoculated in a human gut environment‐simulating growth medium, based on a previously described composition (Fehlbaum et al., 2015; Macfarlane et al., 1998) to obtain a final inoculum concentration of 2% (v/v). The pH of the growth medium was standardized to 6.8 to mimic the human colon pH prior to autoclave (Alessandri et al., 2022a; Macfarlane et al., 1998), while vitamin and mineral solutions were sterilized by filtration using a 0.2‐μm filter and added to culture medium once cooled. Cultivations were carried out in 1 mL of growth medium following the MiPro model (Li et al., 2019a), that is, involving a 96‐deep well plate covered with a silicone gel mat provided with a vent hole on each well, created through a sterile syringe needle. During cultivation, plates were shaken at 500 rpm.

The same protocol was used for the cultivation of vaginal samples. However, in this case, a culture medium mimicking the human vaginal environment, that is, the simulated vaginal fluid, with a pH adjusted to 4.5, as previously described (Pan et al., 2019), was employed. Faecal and vaginal samples were cultivated for 19 and 24 h, respectively, under anaerobic conditions at 37°C. Furthermore, for each of the two matrices, samples were cultured in the presence and absence of 2% α‐LA. After cultivation, all samples were stored at −80°C until they were processed for DNA extraction and bacterial cell enumeration.

### Ethics statement

The study protocol was approved by the Ethical Committee of the IRB Alma Res (Approval number 007/2022) and registered on ClinicaTrials.gov (ClinicalTrials.gov *identifier NCT05674318*). Signed informed consents were obtained from each woman enrolled in the present study and the clinical practice followed the Ethical Principles of the Helsinki Declaration and the national law.

### In vivo evaluation of the effect of α‐lactalbumin on vaginal microbiota of PCOS‐affected women

Ten patients with clinical signs of PCOS were enrolled for the in vivo pilot study at Agunco Obstetric and Gynecologic Centre (Rome, Italy). Specifically, to be enrolled, women had to be on fertile age with a diagnosis of PCOS resulting from (i) menstrual alterations, (ii) polycystic ovary, (iii) biochemical hyperandrogenism (blood levels of testosterone >59 ng/dL or fasting insulin higher than 25 μUI/m) or (iv) phenotypical hyperandrogenism (acne, hirsutism, androgenetic alopecia and seborrhoea). Instead, exclusion criteria included (i) the assumption of antibiotic therapies, prebiotics and/or probiotics in the month prior sample collection; (ii) concomitant inflammatory diseases (celiac disease, Chron's disease); (iii) pregnancy; (iv) physiological and/or induced menopause; (v) neoplastic diseases; (vi) allergy to milk proteins and (vii) the substance abuse (Escobar‐Morreale, 2018; Hong et al., 2021; Witchel et al., 2019). Specifically, all 10 enrolled patients exhibited ultrasound evidence of polycystic ovary and signs of hyperandrogenism. In addition, clinical practice guidelines to correctly collect the vaginal swab were addressed, including no sexual intercourses, no vaginal solution nor ovules in the previous 24 h. To evaluate the possible impact of orally administrated α‐LA on vaginal microbiota in such patients, all the women underwent a 30‐day oral administration of α‐LA (300 mg/twice a day), and two vaginal swabs were collected from each patient: one before (baseline—T0) and one after (T1) α‐LA administration. Immediately after collection, each vaginal swab was transferred into a sterile container with 3 mL of DNA/RNA Shield (Zymo Research, USA) to avoid alteration in the sample and stored at −20°C until processing.

### DNA extraction and Illumina shallow shotgun sequencing

DNA extraction from faecal sample cultivations was performed using the QIAamp Fast DNA Stool Mini Kit (Qiagen, Germany), while the ZymoBIOMICS DNA Miniprep Kit (Zymo Research Corporation, USA) was employed for DNA extraction from both vaginal sample cultivations and vaginal swab collected from the in vivo study. In both cases, DNA extraction was performed following the manufacturer's instructions. Subsequently, the extracted DNA was prepared using the Illumina Nextera XT DNA Library Preparation Kit following the Illumina Nextera XT protocol. Briefly, DNA samples were enzymatically fragmented, barcoded and purified employing magnetic beads. Then, samples were quantified using the fluorometric Qubit quantification system (Life Technologies, USA), loaded on a 2200 Tape Station Instrument (Agilent Technologies, USA) and normalized to 4 nM. A paired‐end sequencing was performed using an Illumina MiSeq sequencer with MiSeq Reagent Kit V3 (Illumina Inc., San Diego, USA).

### Analysis of shallow shotgun microbiome data

The obtained .fastq files were filtered to remove reads with an average quality <25 and sequences of human DNA, while reads with a length of >146 bp were retained. Quality‐filtered data were used for further analysis with the METAnnotatorX2 pipeline for taxonomic profile reconstruction, as previously described (Milani et al., 2021). To evaluate whether significant differences occurred in the taxonomic composition of faecal and vaginal samples before and after α‐LA treatment both in in vitro experiment and in vivo study, a paired Wilcoxon signed‐rank test was employed. Specifically, for each taxon, abundance differences before and after α‐LA treatment were calculated for each subject and then, the obtained differences were subjected to Wilcoxon singed‐rank test calculation. To account for multiple comparisons, the Bonferroni correction was applied to the *p*‐values obtained from the Wilcoxon test. This correction controls the expected proportion of false discoveries among the rejected hypotheses, thus providing a more reliable interpretation of statistical significance. Taxa with an adjusted *p*‐value < 0.05 were considered significantly different between the two considered groups, indicating a significant impact of α‐LA treatment on their abundance.

### Evaluation of bacterial cell density by flow cytometry

For total microbial cell count, each culture/vaginal swab was diluted in physiological solutions (PBS). Subsequently, 1 mL of the obtained bacterial cell suspension was stained with 1 μL of SYBR Green I (ThermoFisher Scientific, USA) (1:100 dilution in dimethylsulfoxide; Sigma, Germany), vortex‐mixed and incubated in the dark for at least 15 min before measurement. All count experiments were carried out using the Attune NxT Flow Cytometry (ThermoFisher Scientific, USA) equipped with a blue laser set at 50 mW and tuned at an excitation wavelength of 488 nm. Multiparametric analyses were performed on both scattering signals, that is, forward scatter (FSC) and side scatter (SSC), while SYBR Green I fluorescence was detected on the BL1 530/30 nm optical detector. Cell debris were excluded from acquisition analyses by setting a BL1 threshold. Furthermore, the gated fluorescence events were evaluated on the forward‐sideways density plot to exclude remaining background events and to obtain an accurate microbial cell count, as previously described (Vandeputte et al., 2017). All data were statistically analysed with the Attune NxT flow cytometry software.

### Statistical analyses

QIIME2 was exploited for the assessment of 16S rRNA microbial profiling‐based α‐ and β‐diversity analyses. To assess the impact of time on the variability between samples while accounting for the paired nature of data in case of in vitro and in vivo pilot study, a permutational multivariate analysis of variance (PERMANOVA) using Bray–Curtis distanc matrices from the taxonomic profile was performed using the ‘adonis’ function from the vegan package (version 2.5–7) through RStudios (http://www.rstudio.com/). Furthermore, to evaluate whether results obtained for the two pooled‐analysis may be affected by bioproject biases, a linear mixed model was employed to analyse data with fixed effects representing the two‐condition group and random effects accounting for the bioprojects. The statistical analysis was performed using the lme4 and dplyr packages using RStudio. SPSS software (www.ibm.com/software/it/analytics/spss) was used to compute the non‐parametric Mann–Whitney *U*‐test and the ANOVA statistics. The false discovery rate (FDR) correction based on Benjamini and Hochberg correction (Benjamini et al., 2001) and calculated using RStudio through ‘*p*.adjust’ function (from base package stats) was applied to statistically significant results.

## RESULTS AND DISCUSSION

### Selection of public data sets

To evaluate possible differences in the taxonomic composition of the intestinal and vaginal microbiota between healthy and PCOS‐affected women, a pooled‐analysis was performed based on publicly available microbiome data sets. Specifically, as a reduced number of studies investigated the faecal and/or vaginal microbiota of women affected by PCOS through shotgun metagenomics, the pooled‐analysis was performed considering only faecal and vaginal samples analysed through 16S rRNA gene microbial profiling to obtain a large number of samples and avoid biases related to the use of different sequencing techniques (Tables 1 and S1). For this purpose, an in‐depth literature search was carried out to retrieve microbiome data sets based on Illumina sequencing technology corresponding to faecal and vaginal samples from both healthy and PCOS‐affected women. In detail, in case of longitudinal studies, only one sample per subject was considered to avoid the inclusion of redundant samples, while for studies encompassing the administration of drugs, hormones, prebiotics and/or probiotics, only faecal and vaginal samples from the pre‐treatment and/or control groups were selected. In addition, to prevent biases associated with other pathologies, only faecal and vaginal samples from women with no diseases other than PCOS were considered. Overall, this literature survey allowed to select a total of 665 publicly available samples, divided into 391 (216 and 175 from healthy and PCOS‐affected women) and 274 (140 and 134 from healthy and PCOS‐affected women) faecal and vaginal samples, respectively (Table S1). Furthermore, to avoid software‐related biases, all samples were re‐analysed through the QIIME2 software pipeline (Bokulich et al., 2018; Caporaso et al., 2010) generating a total of 39,058,882 reads with an average of 58,735 reads per sample, reduced to a total of 29,032,727 reads with an average of 43,658 reads per sample after quality filtering (Table S2).

### Pooled‐analysis of the faecal microbiota of PCOS‐affected women

To investigate possible alterations in the faecal microbial ecosystem correlated to PCOS, the taxonomic composition of the selected publicly available faecal samples from healthy and PCOS‐affected women were compared.

**FIGURE 1:**
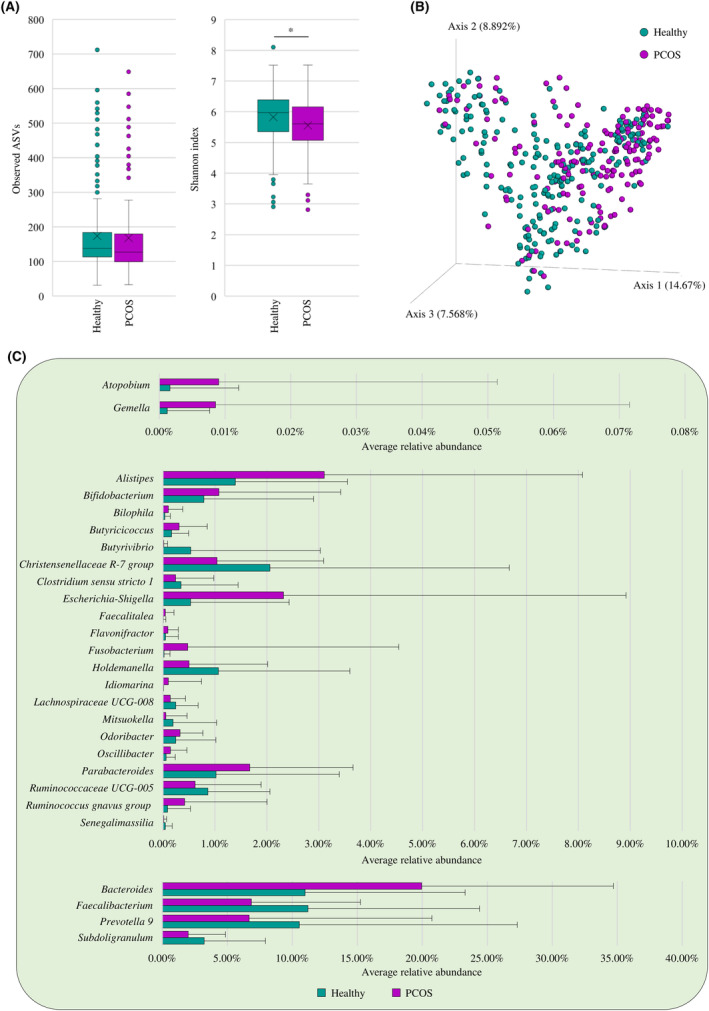
Exploration of the diversity and bacterial composition of the gut microbiota of healthy and PCOS‐affected women. Panel (A) displays the box and whisker plots of the alpha‐diversity calculated through the observed ASV index and the Shannon index of the two clinical groups. The *x*‐axis reports the two considered clinical groups, while the *y*‐axis shows the number of the observed ASVs or Shannon values. Boxes are determined by the 25th and 75th percentiles, while the whiskers are determined by the maximum and minimum values and correspond to the box extreme values. Lines inside the boxes represent the average, while crosses correspond to the median. Panel (B) depicts the three‐dimensional Bray–Curtis dissimilarity index‐based PCoA of the selected faecal samples. Panel (C) highlights the average relative abundance (>0.5%) of the bacterial genera that significantly differ in the gut microbiota of healthy and PCOS‐affected women.

The α‐diversity analysis, calculated both through the observed ASVs and Shannon indexes, revealed a higher complexity of the intestinal microbiota of healthy women when compared to those affected by PCOS, although only the Shannon index highlighted a statistically significant increment (Mann–Whitney *U*‐test *p*‐value of 0.085 and 0.034 for ASVs and Shannon index, respectively) (Figure 1). In parallel, a Bray–Curtis dissimilarity‐based β‐diversity analysis, represented through a PCoA, showed significant compositional differences between the two groups (PERMANOVA *p*‐value < 0.001) (Figure 1). These results suggest that PCOS seems to correlate not only with a reduced intestinal microbial complexity but also with a modulation of the taxonomic composition of the human gut microbiota.

In this context, in‐depth insights into the taxonomic composition of faecal samples from healthy and PCOS‐affected women revealed that the relative abundance of 27 bacterial taxa, including two of the main representative bacterial genera of the human gut microbiota, significantly differed between the two groups, based on the Mann–Whitney *U*‐test corrected for multiple comparisons using the false discovery rate (FDR) (Figure 1A–C and Table S3). Specifically, while the average relative abundance of the genus *Bacteroides* displayed a significant increment in the faecal microbiota of women with PCOS, the genus *Prevotella* showed an opposite trend with a concomitant prevalence reduction (Figure 1 and Table S3). Thus, suggesting that PCOS is strictly associated with an alteration in the average relative abundance of some of the highly abundant microbial genera colonizing the human gut (Arumugam et al., 2011; Costea et al., 2018). At the same time, focusing the attention on those bacterial taxa known to exert either beneficial of detrimental effects upon their host, another abundant and prevalent bacterial taxon, that is, *Faecalibacterium*, showed a twofold increase of the average relative abundance in the faecal samples from healthy women when compared to the PCOS‐affected ones (Mann–Whitney *U*‐test *p*‐value < 0.001) (Figure 1 and Table S3). This microbial genus has attracted particular interest from the scientific community for the ability of certain members of this taxon, that is, *Faecalibacterium prausnitzii*, to exert several beneficial effects upon the host. Indeed, this species not only displays anti‐inflammatory features, but being a butyrogenic microorganism, it also produces butyrate that can be favourably exploited by the intestinal epithelial cells as growth substrate enhancing the epithelial barrier integrity and mucosal immunity (Kazmierczak‐Siedlecka et al., 2022; Lopez‐Siles et al., 2017; Zhang et al., 2022a). Not by chance, *F. prausnitzii* is considered as a biomarker of gut health as its depletion is associated with several pathological disorders (De Filippis et al., 2020; Leylabadlo et al., 2020; Lopez‐Siles et al., 2017), including inflammatory bowel syndrome (IBS) and inflammatory bowel disease (IBD), as well as colorectal cancer and diabetes (Cao et al., 2014). In this context, the reduced abundance of this bacterial taxon in the faecal samples from PCOS‐affected women may be considered as one of the markers for this pathology, also considering that women with PCOS may have a higher risk of developing IBD (Mathur et al., 2010).

Conversely, two bacterial genera encompassing members widely recognized as potential pathogens, that is, *Escherichia‐Shigella* and *Fusobacterium*, possessed a higher abundance and prevalence in the faecal samples of PCOS‐affected women than those from the healthy controls (Figure 1 and Table S3). Specifically, *Fusobacterium*, especially the species *Fusobacterium nucleatum*, has been associated with a plethora of diseases, including IBD and colorectal cancer, for its ability to stimulate a pro‐inflammatory status (Engevik et al., 2021; Gurung et al., 2020; Wong & Yu, 2019). Therefore, the higher abundance of this genus in faecal samples of women with PCOS corroborates its involvement in human diseases and suggests its potential role in contributing to the stimulation of an intestinal inflammatory condition typical of this syndrome (Abraham Gnanadass et al., 2021; Armanini et al., 2022; Gurung et al., 2020; Wong & Yu, 2019). Furthermore, unexpectedly, a bacterial taxon including some members able to exert beneficial effects upon the human host, that is, the genus *Bifidobacterium*, resulted to be more abundant and prevalent in the faecal samples of women with PCOS with respect to the healthy ones (Figure 1 and Table S3). However, the different variable regions of the 16S rRNA gene amplified for the taxonomic profiling of faecal samples among the various studies included in this pooled‐analysis may have influenced such observation. Not by chance, samples obtained from the only two studies that targeted the V1‐V2‐V3 regions (Table 1), known to underestimate the genus *Bifidobacterium* (Mancabelli et al., 2020; Turroni et al., 2009), resulted to be completely depleted of the above‐mentioned taxon (Table S4). Based on this bias, the statistical analysis was recalculated by eliminating the faecal samples of the two bio‐projects. The updated results revealed a slightly higher average relative abundance in the faecal samples from healthy women compared to the PCOS‐affected ones, even though not statistically significant (*p*‐value = 0.790) (Table S4). However, further investigations with a large set of samples analysed with the same primer pair may clarify this hypothesis. Additional detail concerning the evaluation of possible bioproject‐related effects can be found in the Supplementary Text.

Overall, these results highlighted that the gut microbiota of PCOS‐affected women is characterized by a reduced abundance of beneficial microorganisms with a concomitant increment of certain opportunistic pathogens.

### Pooled‐analysis of the vaginal microbiota of women with PCOS

As for faecal samples, to evaluate the correlation between PCOS and changes in the taxonomic composition of the vaginal microbiota, publicly available 16S rRNA gene microbial profiling‐based vaginal samples from healthy and PCOS‐affected women were re‐analysed.

**FIGURE 2:**
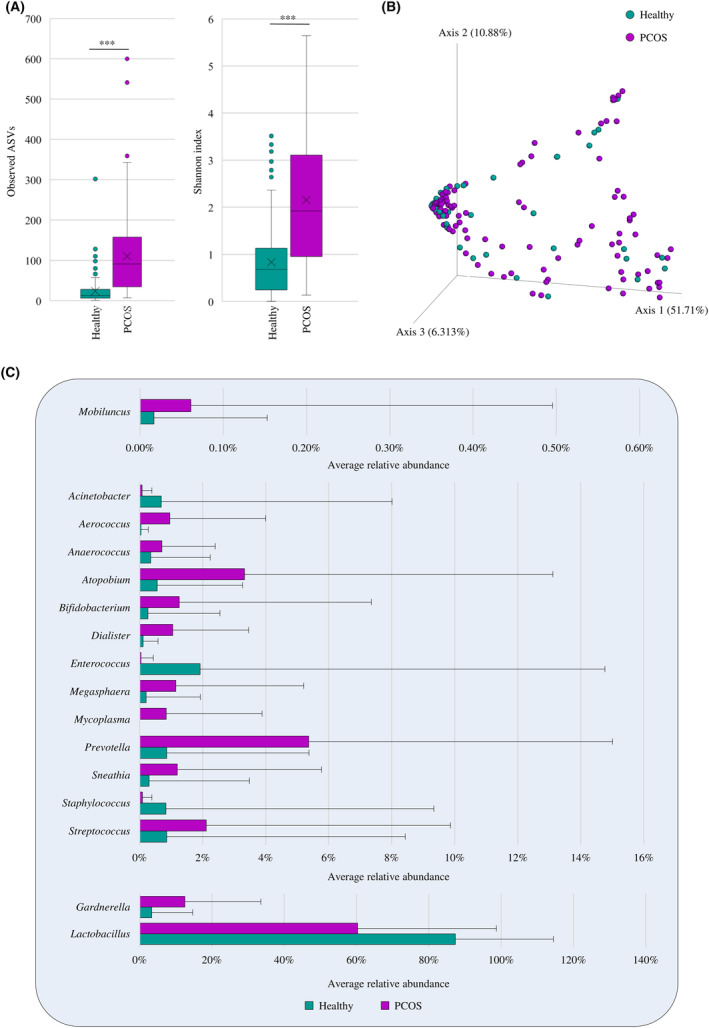
Evaluation of the diversity and bacterial composition of the vaginal microbiota between healthy and PCOS‐affected women. Panel (A) shows the box and whisker plots of the α‐diversity calculated through the observed ASV index and Shannon index of the two clinical groups. The *x*‐axis displays the two considered clinical groups, while the *y*‐axis shows the number of the observed ASVs or Shannon values. Boxes are delimited by the 25th and 75th percentiles, while the whiskers are determined by the maximum and minimum values and correspond to the box extreme values. The crosses inside the boxes correspond to the median, while the lines represent the average. Panel (B) reports the three‐dimensional Bray–Curtis dissimilarity index‐based PCoA of the selected vaginal samples. Panel (C) displays the average relative abundance of the bacterial genera that significantly vary between the vaginal microbiota of healthy and PCOS‐affected women.

The α‐diversity analysis, based on the ASV and Shannon indexes, highlighted that the microbial complexity of the vaginal samples from PCOS‐affected women is significantly higher compared to the healthy ones in which it resulted to be drastically and significantly reduced (Mann–Whitney *U*‐test *p*‐value < 0.001 for both indexes) (Figure 2A–C). This is a common condition to several other urogenital diseases such as bacterial vaginosis, candidiasis and urinary tract or human papillomavirus infections (Deka et al., 2021; Gao et al., 2013; Lev‐Sagie et al., 2022; Liu et al., 2013; Mancabelli et al., 2021b). At the same time, the β‐diversity analysis represented through a PCoA, revealed significant differences in the biodiversity of samples between the two clinical conditions (PERMANOVA *p*‐value < 0.001) (Figure 2). Thus, a significant increase of the complexity of the vaginal microbial ecosystem coupled with a diverse taxonomic composition was observed, indicating drastic alterations in the vaginal microbiota in presence of such syndrome.

In this context, to investigate possible microbial markers associated with PCOS, a non‐parametric Mann–Whitney *U*‐test corrected for multiple correction was applied to the selected samples (Table S5). Interestingly, the average relative abundance of the most representative bacterial genus of the vaginal microbiota, that is, *Lactobacillus*, is markedly reduced in PCOS‐affected women (average relative abundance of 60.30%) when compared to the healthy ones (87.35%) (Figure 2 and Table S5). Such result could be considered as a first clear sign of a dysbiotic vaginal microbiota in case of PCOS, as *Lactobacillus* species generally correlate with a healthy vaginal environment. Indeed, members of this bacterial genus may limit, by competitive exclusion, the proliferation of potential pathogens through the release of bacteriostatic/bactericidal compounds and/or lactic acid production lowering the environmental pH (Argentini et al., 2022; Deka et al., 2021; Fontana et al., 2020; Mancabelli et al., 2021a; Parolin et al., 2021; Scillato et al., 2021). In parallel, the average relative abundance of various potential pathogens commonly involved in vaginal infections, that is, *Atopobium*, *Gardnerella*, *Megasphaera*, *Mycoplasma*, *Mobiluncus* and *Prevotella*, significantly increased in the vaginal microbiota of women with PCOS with respect to the healthy ones (Figure 2 and Table S5) (Abou Chacra et al., 2021; Argentini et al., 2022; Muzny et al., 2020; Saraf et al., 2021). In this context, the drastic reduction of the genus *Lactobacillus* may have induced lactic acid depletion with a concomitant increment of the vaginal environment pH leading to the overgrowth of the above‐mentioned anaerobic bacteria (Muzny et al., 2020; Saraf et al., 2021). Therefore, PCOS results to be characterized by important alterations in the vaginal microbiota towards a dysbiotic status, which is characterized by a disproportional growth of anaerobic pathogens at the expense of members of the health‐related *Lactobacillus* genus.

Overall, these results highlighted profound changes in the vaginal microbiota of women affected by PCOS respect to the healthy ones, with important changes in both complexity and biodiversity of the vaginal microbial ecosystem.

Therefore, considering the observed alterations in both intestinal and vaginal microbiota in women with PCOS when compared to healthy controls, the identification of natural molecules able to restore such dysbiotic condition could be an attractive and useful strategy for improving the management of the syndrome.

### Effect of α‐lactalbumin on *Bifidobacterium* and *Lactobacillus* strain growth performances

α‐LA, a whey globular protein of the mammalian milk, is largely used as dietary supplement not only for its high‐water solubility, heat stability and low immunogenicity in comparison with other milk allergens, but also for its multiple beneficial effects. Indeed, bioactive peptides generated from the α‐LA proteolytic digestion exert antibacterial, anti‐inflammatory, analgesic, antihypertensive and immunomodulatory actions (Boscaini et al., 2019; Cardinale et al., 2022; Chen et al., 2022; Li et al., 2019b; Zhang et al., 2022b). Recently, α‐LA has been proposed as a compound with prebiotic features able to prevent or recover intestinal dysbiosis inducing an increment in the relative abundance of certain beneficial microorganisms such as *Bifidobacterium* and *Lactobacillus* strains (Boscaini et al., 2019; Cardinale et al., 2022; Chen et al., 2022; Li et al., 2019b). In this context, as the pooled‐analysis highlighted that PCOS correlates with a reduced abundance of the genera *Bifidobacterium* and *Lactobacillus* in the intestinal and vaginal microbial ecosystem, respectively, the possible positive influence of α‐LA on the growth performances of members of the two above‐mentioned bacterial taxa was evaluated. Specifically, 17 *Bifidobacterium* and 11 *Lactobacillus* strains isolated from human faecal and vaginal samples, respectively, were grown in presence of three different concentrations of α‐LA, that is, 1%, 2% and 3%, as well as in the absence of this or other additional proteins (control sample) (Table 2). In detail, the strains were selected to test at least one strain for each of the most representative *Bifidobacterium* and *Lactobacillus* species of the human intestinal and vaginal microbiota, respectively (Derrien et al., 2022; Mancabelli et al., 2021b). Furthermore, an outlier for each bacterial genus was included in the analysis, that is, *Lactobacillus johnsonii* DSM 20533 and *Bifidobacterium asteroides* LMG 10735.

**FIGURE 3:**
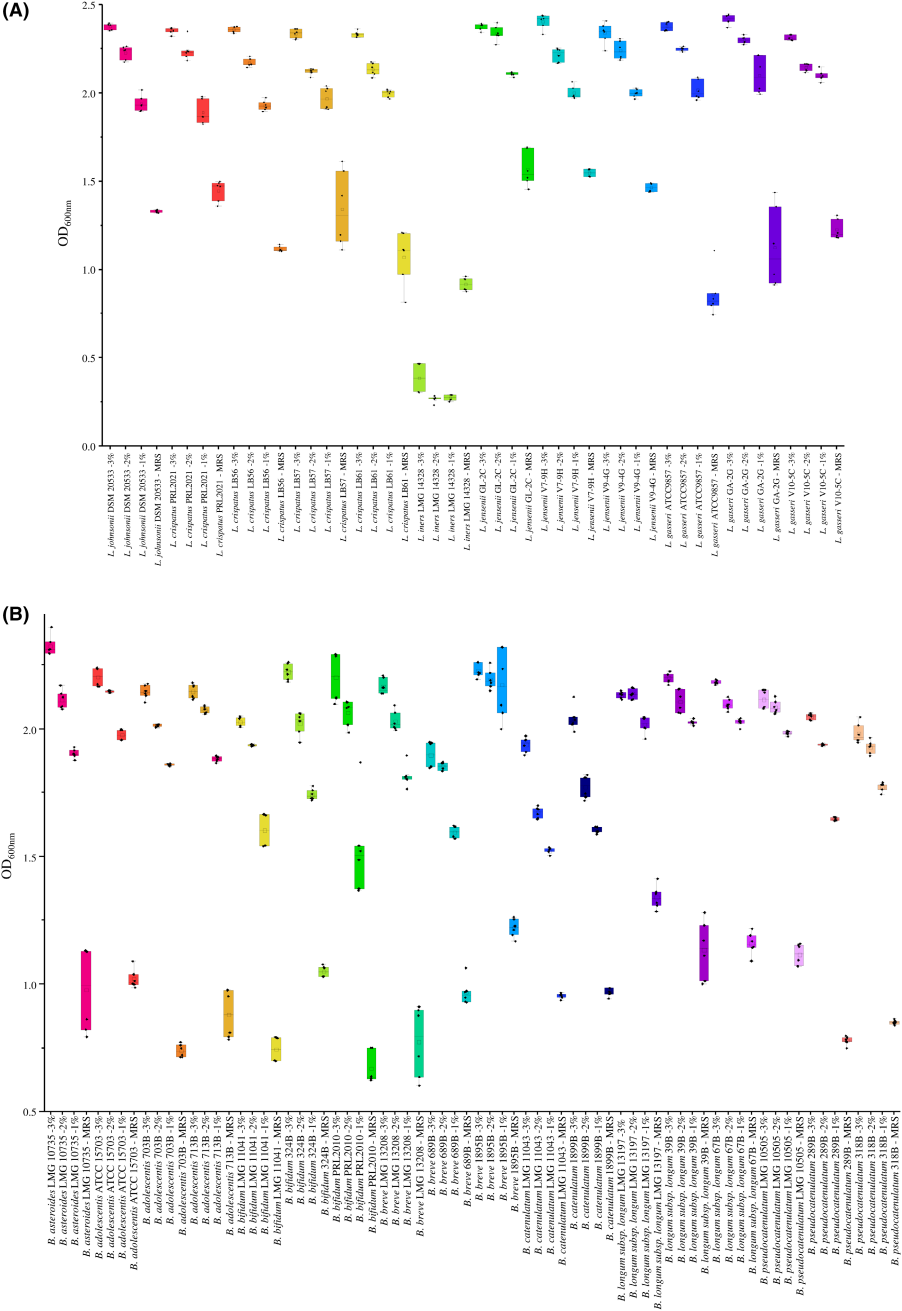
Growth performances of *Bifidobacterium* and *Lactobacillus* strains on α‐LA. Panels (A) and (B) show the box and whisker plots of the growth performances of *Bifidobacterium* and *Lactobacillus* strains, respectively, measured as OD_600nm_. The *x*‐axis identifies the bacterial strains and the α‐LA concentration (1%, 2% and 3%) coupled with the control condition (MRS without the addition of α‐LA), while the *y*‐axis displays the OD_600nm_ measure. Boxes are determined by the 25th and 75th percentiles. The whiskers are delimited by the maximum and minimum values and correspond to the box extreme values. Lines inside the boxes represent the average, while the squares indicate the median.

Interestingly, all tested strains displayed significant higher growth performances when cultivated for 48 h on α‐LA if compared to the control condition, except for *L. iners* LMG 14328 that showed an opposite trend. Specifically, the microbial growth seemed, in almost all cases, to increase significantly with increasing α‐LA concentration (one‐way ANOVA *p*‐value < 0.05) (Figure 3A,B and Table S6). This suggests that this milk protein may act as a prebiotic growth substrate for members of the genus *Bifidobacterium* and *Lactobacillus* enhancing their growth. However, although an increase in growth performances was observed for each strain, only eight strains, that is, *Lactobacillus gasseri* ATCC 9857, *Bifidobacterium adolescentis* 703B, *B. adolescentis* 713B, *Bifidobacterium bifidum* LMG 11041, *B. bifidum* PRL2010, *Bifidobacterium breve* LMG 13208, *Bifidobacterium pseudocatenulatum* 289B and *B. pseudocatenulatum* 318B, exhibited at least a twofold OD_600nm_ increment when cultivated on MRS supplemented with any of the three tested α‐LA concentrations with respect to the control condition (Figure 3). This result indicated that the ability to use α‐LA as a growth substrate is strain specific. Probably, depending on their genetic makeup, some strains can degrade α‐LA into a wider range of peptides and/or amino acids, which can be favourably exploited as additional growth substrate.

Of note, while all tested bifidobacterial strains, except for *B. longum* LMG 13197, exhibited an increase in growth performances of almost twofold with 3% α‐LA with respect to the control, *Lactobacillus* strains displayed a variable behaviour depending on the species. Indeed, only *Lactobacillus crispatus* and *L. gasseri* strains exhibited a 2‐fold OD_600nm_ increase in presence of 3% α‐LA, while *L. iners* and *L. jensenii* strains displayed reduced growth performances (Figure 3). Considering that several evidence report *L. crispatus* and *L. gasseri* as positive microbial biomarkers associated with vaginal health, the ability of α‐LA to favour their growth strengthens the assumed prebiotic features of such protein (Argentini et al., 2022; Chee et al., 2020; Fontana et al., 2020; Tachedjian et al., 2017). At the same time, the inhibition of *L. iners* suggests a possible use of α‐LA as a compound to limit the growth of this controversial *Lactobacillus* species. Indeed, despite its dominance in the vaginal microbiota of a large number of healthy women, it may also act as an opportunistic pathogen participating in a high number of bacterial vaginosis (Sabbatini et al., 2021; Zheng et al., 2019, 2021). As a matter of fact, a vaginal microbiota dominated by *L. iners* offers less protection against vaginal dysbiosis and the occurrence of bacterial vaginosis, sexually transmitted infections (including human papilloma virus infections) and adverse pregnancy outcomes (Mitra et al., 2016; Zheng et al., 2021). However, a larger number of *L. iners* strains are required to validate this hypothesis.

Overall, these results highlighted that α‐LA may be a valid compound to be administrated with the aim to favour the growth of beneficial bacteria such as members of the genera *Bifidobacterium* and *Lactobacillus* that are able to use such molecule as a prebiotic substrate.

### In vitro evaluation of α‐lactalbumin impact on female intestinal and vaginal microbiota

**FIGURE 4:**
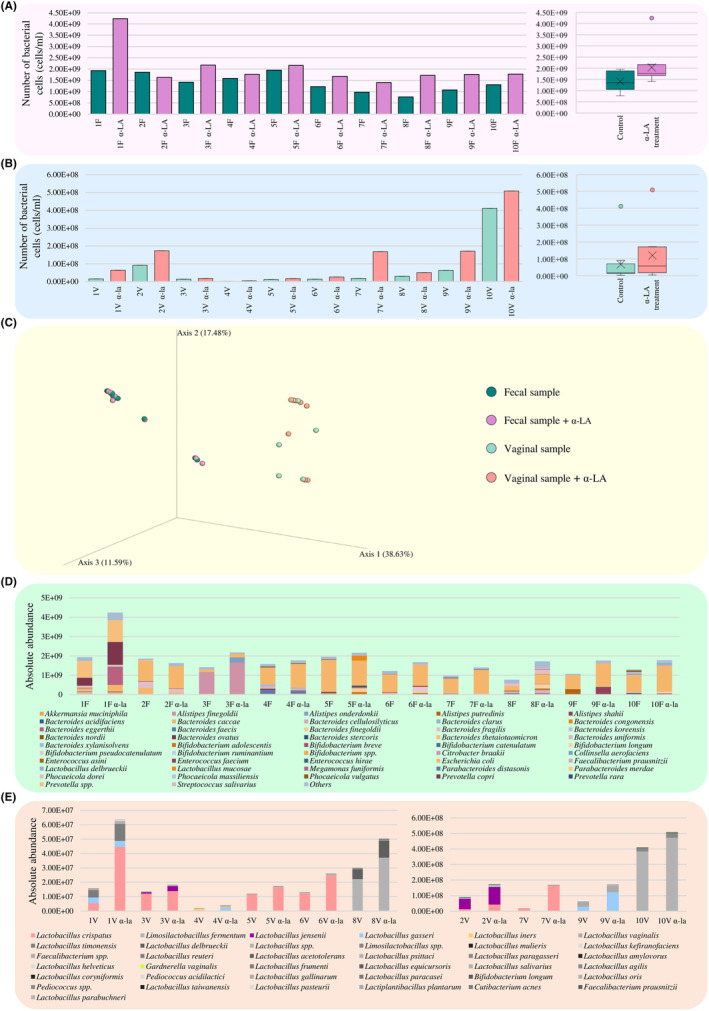
Effect of α‐LA on female intestinal and vaginal microbiota. Panel (A) and (B) show bar plots (on the left) and box and whisker plots (on the right) related to the bacterial cell enumeration of each cultivated intestinal and vaginal sample, respectively, in the presence and absence of α‐LA and the average of the bacterial counts per group. Panel (C) reports the three‐dimensional Bray–Curtis dissimilarity index‐based PCoA associated with the taxonomic profiles of both intestinal and vaginal microbiota of 10 healthy women cultivated with and without α‐LA. Panel (D) and (E) display the taxonomic profiles obtained through shallow shotgun sequencing after culturing intestinal and vaginal samples, respectively, from 10 healthy women in presence and absence of α‐LA. Only bacterial species cited in the text were highlighted.

To investigate whether α‐LA may have a positive impact also on complex microbial community, faecal and vaginal samples collected from 10 healthy women were cultivated in a human intestinal and vaginal environment‐simulating growth media for 19 and 24 h, respectively, in presence and absence of 2% α‐LA. In detail, although the prebiotic effect of α‐LA seemed to increase with increasing concentration, as demonstrated above for members of the genera *Bifidobacterium* and *Lactobacillus*, for the following in vitro experiments, a concentration of 2% α‐LA was selected to mimic carbohydrate‐based growth assays that generally test the bacterial ability to grow on a specific glycan substrate with a concentration ranging from 0.5% to 2% (Alessandri et al., 2022a; Lugli et al., 2019; McLaughlin et al., 2015; Watson et al., 2013). After cultivation, growth performances of each sample were assessed through flow cytometry‐based bacterial cell enumeration. Interestingly, the number of bacterial cells was significantly higher in presence of α‐LA for both faecal (average bacterial count of 2.03E+09 cells/mL) and vaginal (average bacterial count of 1.20E+08 cells/mL) samples when compared to the control groups (average bacterial count of 1.41E+09 cells/mL and 6.70E+07 cells/mL for faecal and vaginal samples, respectively) (paired Wilcoxon test with Bonferroni correction *p*‐value < 0.01for both samples types) (Figure 4A–E and Table S7). Thus, suggesting that this protein may have a role in improving the growth performances of certain bacterial taxa.

Furthermore, to evaluate whether the exposure to α‐LA may also play a role in the modulation of the taxonomic composition of intestinal and vaginal microbiota, the above‐mentioned cultures were also subjected to DNA extraction and species‐level taxonomic profiling through shallow shotgun metagenomics. The sequencing resulted in a total of 4,521,317 reads, reduced to 2,319,965 reads with an average of 57,999 reads per sample after quality filtering (Table S8).

As expected, a Bray–Curtis dissimilarity‐based β‐diversity analysis revealed a clear separation of samples based on their origin, that is, faecal or vaginal samples (Figure 4B). However, neither the faecal nor the vaginal control samples clustered separately from the corresponding α‐LA‐treated samples (Figure 4B). Thus, suggesting that the exposure to α‐LA did not induce any significant drastic change in the biodiversity of samples, as observed by the calculation of PERMANOVA using the Adonis function (*R* ^2^ of 0.01023 and 0.0031 for faecal and vaginal samples, respectively, and PERMANOVA *p*‐value >0.05 for both sample types). An observation that can be considered as a positive factor since an excessive bacterial upheaval could cause dysbiosis. Indeed, considering that the analysed microbial samples derived from healthy individuals, observing that α‐LA administration does not subvert the architecture of the microbiota, instead it rebalances some alterations, could be an advantageous aspect of safety towards the microbiota physiology.

In addition, although in depth insights into the taxonomic profiles revealed no significant differences neither for faecal nor for vaginal samples between the control and treated group (paired Wilcoxon test with Bonferroni correction *p*‐value >0.05 for all comparisons) (Table S9), probably due to the high heterogenicity of the samples, the analysis of the sequencing data highlighted interesting trend with members of the most representative bacterial genera of the human gut microbiota, that is, *Alistipes*, *Bacteroides*, *Parabacteroides* and *Phocaeicola* that underwent a decrease in relative abundance if cultivated in presence of *α*‐LA when compared to the control (Figure 4C and Table S9) (Arumugam et al., 2011; Mancabelli et al., 2017). On the other side, although with some exceptions, species of the genus *Bifidobacterium*, including *B. adolescentis*, *B. breve*, *Bifidobacterium catenulatum*, *B. longum*, *B. pseudocatenulatum* and *Bifidobacterium ruminantium* displayed an increased relative abundance in faecal samples grown in the presence of *α*‐LA when compared to the control (Figure 4C and Table S9). In this context, as certain members of the genus *Bifidobacterium* are well recognized as microorganisms exerting multiple beneficial effects upon their host, their observed higher relative abundance in the faecal samples cultivated with *α*‐LA indicates that this protein may have a bifidogenic effect, also confirming the above‐mentioned prebiotic feature of *α*‐LA towards members of the genus *Bifidobacterium* (Alessandri et al., 2021; Bottacini et al., 2017; Hidalgo‐Cantabrana et al., 2017). Interestingly, beyond bifidobacteria, also *Prevotella copri*, a bacterial species currently described as a new generation probiotic, displayed an increased relative abundance when exposed to α‐LA, leading to assume that this protein may exert a prebiotic effect also stimulating the growth of this new generation probiotic (Figure 4D and Table S9) (Abenavoli et al., 2020; De Filippis et al., 2022).

Similarly, the analysis of data related to the growth performances of vaginal samples in presence and absence of *α*‐LA revealed alterations in the main bacterial representatives of the vaginal microbiota, even if not statistically significant (paired Wilcoxon test with Bonferroni correction *p*‐value >0.05 for all comparisons) (Figure 4D and Table S9). Interestingly, in all the vaginal samples with a high relative abundance (>10%), except for sample 3, *L. crispatus* showed better growth performances in presence of *α*‐LA (Figure 4D and Table S9). This suggests the ability of the tested protein to stimulate the growth of this healthy vaginal environment‐associated microbial taxon, thus corroborating results on single strains of *L. crispatus* on *α*‐LA. Conversely, *L. iners* displayed a reduced relative abundance in vaginal samples grown on *α*‐LA when compared to the control, except for sample 2 in which this species is mostly stable between the control and the treated sample (Figure 4D and Table S9). Interestingly, the vaginal sample 4, when cultivated in absence of *α*‐LA resulted to be dominated by *L. iners* whose relative abundance drastically decreased in presence of *α*‐LA, favouring the overgrowth of *L. gasseri*, one of the *Lactobacillus* species generally abundant in the vaginal microbiota of healthy women (Argentini et al., 2022; Ravel et al., 2011). In this context, although it represents a dominant species of the vaginal microbiota as *L. crispatus*, the role of *L. iners* is controversial. Plenty of evidence reported a crucial role of *L. iners*‐dominated microbiota in predisposing the ecological niche to pathogen invasion. Indeed, even if it is considered as a common symbiont of the vaginal microbiota of healthy women, it may also act as an opportunistic pathogen participating in a high number of bacterial vaginosis (Mancabelli et al., 2021b; Sanozky‐Dawes & Barrangou, 2022; Zheng et al., 2021) or correlating with the occurrence of sexually transmitted infections as in the case of human papilloma virus (Zeng et al., 2022). Therefore, these data suggest that *α*‐LA may have a role in maintaining stable/limiting the proliferation of *L. iners* and thus avoiding the occurrence of some adverse clinical conditions related to this bacterial species. Similarly, *α*‐LA did not seem to favour the growth of another opportunistic pathogen of the vaginal microbiota, that is, *Gardnerella vaginalis*, whose relative abundance remained stable between the control and treated sample (Figure 4D and Table S9). However, this species was present in only one of the 10 analysed vaginal samples. Therefore, a higher number of samples comprising *G. vaginalis* is required to validate this hypothesis.

Overall, for both faecal and vaginal samples, *α*‐LA seems to exert a prebiotic effect for certain positive and health‐promoting species of the intestinal and vaginal microbiota, including members of the genera *Bifidobacterium* and *Lactobacillus*, while limiting the growth of (possible) opportunistic pathogens.

### Effect of α‐lactalbumin on the vaginal microbiota of women with PCOS

Based on the in vitro results, an in vivo pilot study further evaluated the positive impact of the administration of *α*‐LA on the vaginal microbiota of women affected by PCOS. Specifically, 10 women with clinical signs of PCOS were enrolled and privately followed at Agunco Obstetric and Gynecologic Centre (Rome, Italy), following the ethical principles of the Helsinki Declaration and the national law.

**FIGURE 5:**
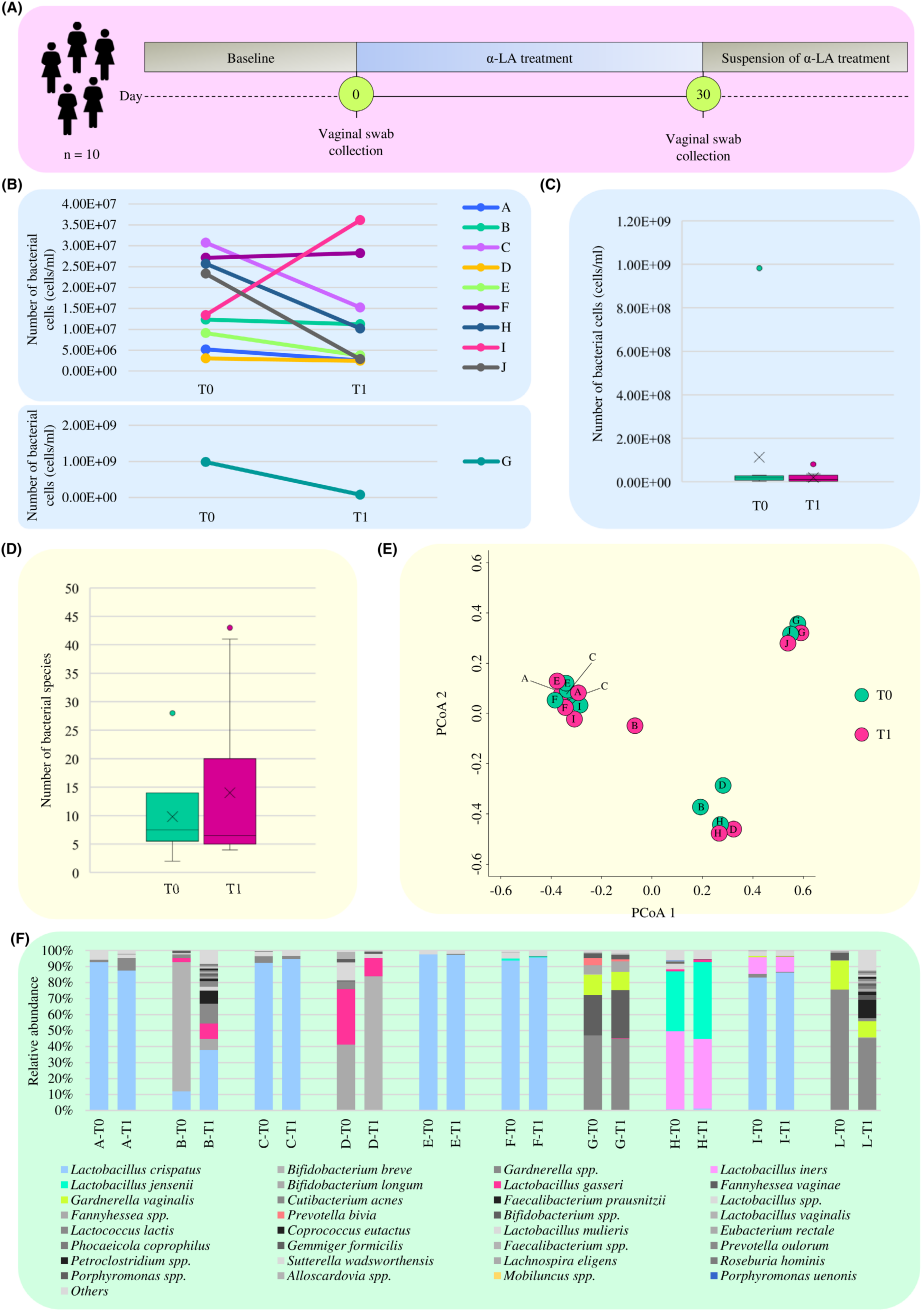
α‐LA impact on the vaginal microbiota of PCOS‐affected women. Panel (A) shows the time line of the experimental procedures of the in vivo pilot study. Panel (B) displays the flow cytometry‐based bacterial cell count of the vaginal swab collected from the 10 women enrolled in the in vivo pilot study at time points T0 and T1, while panel (C) reports the box and whisker plot of the average of the bacterial cell count per considered time points. The *x*‐axis shows the two time points, while the *y*‐axis reports the number of bacterial cells. Panel (D) depicts the box and whisker plot of the average number of bacterial species detected in the collected vaginal swabs divided per time points. The *x*‐axis shows the two time points, while the *y*‐axis reports the number of bacterial species. In both box and whisker plots, boxes are determined by the 25th and 75th percentiles. The whiskers are determined by the maximum and minimum values and correspond to the box extreme values. Lines inside the boxes represent the average, while crosses correspond to the median. Panel (E) depicts the three‐dimensional Bray–Curtis dissimilarity index‐based PCoA of the 20 vaginal swabs collected before and after 30 days of α‐LA administration, while panel (F) reports the shallow shogun‐based taxonomic profiles of the vaginal microbiota of the 10 women suffering from PCOS before and after α‐LA treatment.

All the enrolled women exhibited at least two of the three Rotterdam Criteria for the diagnosis of PCOS (Rotterdam, 2004), in particular phenotypical hyperandrogenism and ultrasound evidence of polycystic ovaries. All patients were orally administered with *α*‐LA (300 mg twice a day) for 30 days, and for each subject a vaginal swab was collected before (T0—baseline) and after (T1) *α*‐LA treatment (Figure 5A).

To evaluate whether the administration of *α*‐LA may have a role in modulating the total bacterial load of the vaginal microbiota, the vaginal swabs were subjected to a flow cytometry‐based total bacterial cell count. In detail, in all cases, except for samples F, I and L, a higher total bacterial count was recorded at T0 when compared to T1 (Figure 5B and Table S10), suggesting that the administration of this milk‐derived protein may in vivo reduce the bacterial load of the vaginal microbiota. However, although the average total bacterial cell count at T0 was almost 10 times higher than that at T1 (average bacterial cell count of 1.13E+08 cells/mL and 1.93E+07 cells/mL, respectively) (Figure 5C), the observed difference was not statistically significant (paired Wilcoxon test with Bonferroni correction *p*‐value = 0.105), probably due to the high heterogeneity of the collected samples in terms of total bacterial count.

Furthermore, to assess whether the administration of *α*‐LA may modulate the taxonomic composition of PCOS‐affected women vaginal microbiota, the 20 collected vaginal swabs were subjected to a taxonomic profile using shallow shotgun sequencing, generating a total of 142,935 filtered reads with an average of 7147 reads per sample (Table S11). Based on the obtained taxonomic results, the species richness analysis revealed no significant differences in the average number of species detected in the vaginal samples of women suffering from PCOS between T0 and T1 (Mann–Whitney *U*‐test *p*‐value = 0.909) (Figure 5D). Similarly, a Bray–Curtis dissimilarity‐based β‐diversity analysis represented through a PCoA did not show significant changes in the vaginal bacterial composition between T0 and T1 (*R* ^2^ = 0.00764 and PERMANOVA *p*‐value >0.05) (Figure 5E). Thus, suggesting that, as observed for total bacterial load, *α*‐LA administration does not induce drastic/major shifts either in the species richness or biodiversity of the vaginal microbiota of women suffering from PCOS. However, although no appreciable alterations were observed in the biodiversity of the vaginal microbiota of PCOS‐affected women after 30 days of *α*‐LA treatment, possible differences in the taxonomic profiles between T0 and T1 were investigated. In detail, although no significant differences emerged in the average relative abundance of the detected bacterial species between T0 and T1 (paired Wilcoxon test with Bonferroni correction *p*‐value >0.05 for all species), certain trends were observed between the two examined time points. Interestingly, the relative abundance of *L. crispatus*, when present, remained stable (for samples A and E) or even increased (for samples B, C, F, H and I) (Figure 5F). In this context, as this species is considered as a crucial positive microbial biomarker in the vaginal environment due to its claimed beneficial implications on vaginal health (Argentini et al., 2022; Chee et al., 2020; Mancabelli et al., 2021a), it can be assumed that the intake of *α*‐LA can have a positive effect on the host health favouring the persistence of this species in the vaginal environment. Similarly, *L. jensenii* and *L. gasseri*, other two predominant *Lactobacillus* species generally related to a healthy vaginal microbiota, as also above‐mentioned (Mancabelli et al., 2021b), displayed an increased relative abundance in T1 respect to T0, except for sample F and D, respectively, where the two species showed an opposite trend (Figure 5F). These data indicate that *α*‐LA may have a beneficial effect favouring an increase in the abundance of health‐promoting *Lactobacillus* species, even if with some exceptions. On the other side, in both samples in which it was detected, the opportunistic pathogen *L. iners* underwent a slight decrease in its relative abundance at T1 (Figure 5F), suggesting that *α*‐LA may act maintaining/limiting the abundance of this controversial species potentially preventing its overgrowth and confirming the above obtained in vitro results (Mancabelli et al., 2021b; Zheng et al., 2021).

In parallel, the opportunistic pathogen *G. vaginalis* showed, in all three cases in which it was present, a relative abundance reduction after 1 month of *α*‐LA administration, denoting that this protein may have a role in limiting the growth and proliferation of this pathogenic bacterium. Similarly, even if present in a single sample, *Prevotella bivia*, another pathogenic microorganism strictly associated with vaginal infection, showed a decreased relative abundance after α‐LA intake (Figure 5F). In addition, even if they showed a reduced relative abundance (<1%), members of the genus *Mobiluncus* and *Porphyromonas uenonis* generally known as opportunistic pathogens of the vaginal tract, underwent a relative abundance reduction in sample H at T1 respect to T0 (Figure 5F) (Argentini et al., 2022; Barczynski et al., 2023; Mohankumar et al., 2022). Overall, these results allowed for the first time to hypothesize that this protein may have a role in reducing the abundance/limiting the growth of potential pathogens while favouring the proliferation of health‐promoting bacteria not only in in vitro but also in in vivo conditions. Up to date, only a few studies evaluated the effects of an oral supplementation on vaginal microbiota composition and, as far as we know, they all concerned probiotics rather than natural molecules with prebiotic activity, as the α‐LA (Ansari et al., 2023; Reid et al., 2001, 2003; Yang et al., 2020). Overall, these results not only corroborate the potential prebiotic role of this milk‐derived protein in treating the microbial alterations in the vaginal microbiota typical of PCOS‐affected women, but they also allow this molecule to be nominated as a possible strategy to alleviate/limit the dysbiotic condition associated with PCOS, regardless of phenotypes or hyperandrogenic/norm androgenic profile of the PCOS‐affected patients.

Of course, in our study a higher number of samples is needed to achieve statistical power and to validate the potential beneficial effect of α‐LA in modulating the vaginal microbiota of women with PCOS towards a healthy microbiota. However, considering the alterations in the intestinal and vaginal microbiota typical of PCOS‐affected women, strengthening the potential prebiotic role of the α‐LA could pave the way for improving the management of the syndrome by restoring dysbiotic microbiota.

## CONCLUSIONS

Considering the impact of PCOS on both life quality and health, in the last decades, the scientific community has focused particular attention on understanding mechanisms triggering this syndrome with the aim to identify potential solutions to prevent/treat PCOS symptoms. However, the hypothesis of the involvement of intestinal and vaginal microbiota in the onset of the syndrome has been postulated only in recent years. Indeed, still little evidence highlight the association between the intestinal/vaginal microbial ecosystems and PCOS. In this context, our pooled‐analysis, performed by re‐analysing the publicly available 16S rRNA microbial profiling of faecal and vaginal samples from healthy and PCOS‐affected women, shed light on the possible correlation between variations in gut and vaginal microbiota and PCOS. This analysis revealed that this syndrome was characterized by a dysbiotic condition with a reduction of beneficial bacteria, including *Bifidobacterium* and *Faecalibacterium* for the gut microbiota and *Lactobacillus* for the vaginal environment, and a concomitant enrichment of potential pathogens, encompassing *Escherichia* and *Fusobacterium* for the intestinal microbial ecosystem and *Atopobium*, *Gardnerella*, *Megasphaera*, *Mycoplasma*, *Mobiluncus* and *Prevotella* for the vaginal microbiota.

Furthermore, the in vitro evaluation of the potential ‘prebiotic’ feature of α‐LA highlighted its ability to improve the growth performances of members of two health‐promoting bacterial genera that are depleted in the intestinal and vaginal microbiota of PCOS‐affected women, that is, *Bifidobacterium* and *Lactobacillus*, respectively, and to modulate the taxonomic composition of both gut and vaginal microbial ecosystems. Finally, an in vivo pilot study based on the oral supplementation of α‐LA for 30 days to women suffering from PCOS corroborated results from the in vitro experiments, revealing that α‐LA administration seemed to maintain stable/favour the overgrowth of health‐promoting bacteria, including *L. crispatus*, *L. gasseri* and *L. jensenii*, while limiting the growth of potential vaginal pathogens. However, the results obtained from the enumeration of bacterial cells in vaginal samples from the in vitro and in vivo experiments were in contrast. Indeed, while a significant increase in the number of bacterial cells was recorded in case of in vitro cultivation of vaginal samples with α‐LA when compared to the control samples, in the in vivo pilot study, even if not statistically significant, an opposite trend was observed. Probably, while in the former case the milk‐derived protein was added directly to the medium and was, therefore, completely bioavailable as a prebiotic substrate thus possibly favouring bacterial growth, in the latter case several variables come into play preventing knowing the real concentration of protein that reaches the different human districts and whether if this concentration varies among individuals. Naturally, also the low numbers of enrolled individuals and the short term of the analysis may represent limitations to these obtained preliminary clinical results.

Overall, these results emphasized the strict association between PCOS and a dysbiotic intestinal and vaginal microbiota, when compared to those from healthy women, thus also strengthening the hypothesis that these two microbial ecosystems are involved in the pathogenesis of this syndrome, confirming the DOGMA theory previously proposed when considering PCOS. Therefore, besides reinforcing the importance of taking into account intestinal and vaginal microbial alterations in the management of PCOS, these results also highlight, for the first time, the potential prebiotic activity of α‐LA in restoring/limiting the dysbiotic intestinal/vaginal environment associated with this syndrome. Indeed, obtained results suggest that α‐LA may play a role in limiting PCOS‐related microbial alterations in the vaginal and gut microbiota, regardless of PCOS phenotypes or hyperandrogenic/norm androgenic profile and in promoting the growth of bacteria with beneficial effects on the human host. Certainly, these preliminary data should be confirmed with further analyses and supporting results, including a larger number of samples for both in vitro experiment and in vivo study to provide mechanistic inside into the role of α‐LA. In addition, the lack of data related to possible changes that the intestinal, and not only the vaginal, microbiota may undergo in women suffering from PCOS after α‐LA treatment, represents a limitation of the present study, but at the same time a suggestion for future designed studies. Indeed, the inclusion of this data, along with the evaluation of the possible modulation of PCOS‐related symptoms after α‐LA treatment, would have allowed to obtain a global vision of the impact that this protein can have on the dysbiotic intestinal and vaginal microbiota associated with PCOS, possibly validating the obtained in vitro results. At the same time, a pilot clinical study that does not exclusively evaluate how the taxonomic composition of the vaginal microbiota is modulated by the administration of α‐LA, but also the possible evolution of PCOS‐associated symptoms would provide higher consistency to the beneficial effect provided by this milk‐derived protein in PCOS management. Finally, further studies aimed at evaluating whether the vaginal administration of α‐LA can have higher positive effects than oral administration are necessary to define the best route of α‐LA administration in the management of PCOS.

However, the up‐to‐date recognized ability of α‐LA to improve the absorption of inositols, that is, natural compounds largely used in the management of PCOS, in case of inositol resistance (Cardinale et al., 2022; Hernandez Marin et al., 2021; Kamenov et al., 2023; Lagana et al., 2024; Montanino Oliva et al., 2018; Ranaldi et al., 2020), combined with the obtained results highlighting the potential beneficial effects of this protein in limiting/restoring the vaginal and intestinal dysbiosis typical of the this syndrome, suggest α‐LA as a promising treatment for PCOS.

## AUTHOR CONTRIBUTIONS

**Giulia Alessandri:** Conceptualization; investigation; writing – original draft; formal analysis; data curation; visualization. **Leonardo Mancabelli:** Conceptualization; methodology; software; formal analysis; validation; investigation; data curation. **Federico Fontana:** Software; formal analysis; data curation. **Elisa Lepore:** Conceptualization; supervision; writing – review and editing; resources. **Gianpiero Forte:** Conceptualization; supervision; resources; writing – review and editing. **Moira Burratti:** Methodology. **Marco Ventura:** Writing – review and editing; funding acquisition; conceptualization; supervision; resources. **Francesca Turroni:** Writing – review and editing; project administration; conceptualization; supervision; resources; funding acquisition.

## CONFLICT OF INTEREST STATEMENT

E.L. and G.F. are employee at LoLi pharma SRL. The other authors declare no competing interest.

## Supporting information


Data S1.

Tables S1–S12.

## Data Availability

The data that support the findings of this study are openly available in NCBI SRA at https://www.ncbi.nlm.nih.gov/sra, reference number PRJNA1075117.
