# Supplementary material for: Disclosing α‐lactalbumin impact on the intestinal and vaginal microbiota of women suffering from polycystic ovary syndrome
Source: Microb Biotechnol. 2024 Oct 4;17(10):e14540. doi: 10.1111/1751-7915.14540 (PMC11450379; doi:10.1111/1751-7915.14540)
Supplement: Supplementary file 1 — Data S1. [file MBT2-17-e14540-s001.docx]

**Disclosing α-lactalbumin impact on the intestinal and vaginal microbiota of women suffering from polycystic ovary syndrome**

Running title: α-lactalbumin effect on female fecal and vaginal microbiota

Key words: α-lactalbumin, shallow shotgun, flow cytometry, PCOS, therapeutic strategies, *Bifidobacterium*, *Lactobacillus*

Giulia Alessandri^1^, Leonardo Mancabelli^2,3^, Federico Fontana^1^, Elisa Lepore^4^, Gianpiero Forte^4^, Moira Burratti^5^, Marco Ventura^1,3^, Francesca Turroni^1,3^

^1^Laboratory of Probiogenomics, Department of Chemistry, Life Sciences, and Environmental Sustainability, University of Parma, Parma, Italy; ^2^Department of Medicine and Surgery, University of Parma, Parma, Italy; ^3^Microbiome Research Hub, University of Parma, Parma, Italy; ^4^R&D Department, Lo.Li Pharma, Rome, Italy; ^5^A.G.Un.Co. Obstetrics and Gynaecology Center, Rome, Italy

Correspondence

Mailing address for Francesca Turroni, Laboratory of Probiogenomics, Department of Chemistry, Life Sciences, and Environmental Sustainability, University of Parma, Parco Area delle Scienze 11a, 43124 Parma, Italy. Phone: ++39-521-906644. Fax: ++39-521-905604. E-mail: francesca.turroni@unipr.it

**Pooled-analysis of the fecal and vaginal microbiota of women suffering from PCOS.** To evaluate whether bioproject-specific variations may affect the significance of certain bacterial taxa, a linear mixed model approach was applied by considering only those bacterial species that significantly differed between the control and PCOS groups and by incorporating PCOS and bioprojects as fixed and random variables, respectively. Interestingly, this analysis highlighted extremely low standard deviation values, thus suggesting that the significant results obtained from the application of the Mann-Whitney U test on the taxonomic composition of both fecal and vaginal samples between healthy and PCOS-affected women should not be affected by bioproject bias (Table S12). Furthermore, through the calculation of the t-value for the fixed variable, i.e., the bacterial genera, this analysis also allowed to evaluate whether a bacterial genus positively or negatively correlates with PCOS. Specifically, only those species with a t-value either > 2 or <-2 were considered as statistically significant. Interestingly, the analysis confirmed what was previously observed through the application of the Mann-Whitney U test between healthy and PCOS-affected women. In detail, the genus *Lactobacillus* was the only bacterial taxon that negatively correlated with PCOS with a t-value < -5. Thus, confirming that this syndrome is characterized by a reduced abundance of this commensal genus of the vaginal environment. In parallel, various vaginal pathogens, i.e., *Gardnerella*, *Ureaplasma*, *Aerococcus*, *Prevotella*, *Atopobium*, and *Mycoplasma*, resulted to be strictly correlated (t-value > 2) with a PCOS condition, strengthening the notion that this syndrome is generally accompanied by an overgrowth of opportunistic pathogens and, therefore, by vaginal dysbiosis. Similarly to vaginal samples, also for fecal microbiota, certain bacterial genera showed significant differences between healthy and PCOS-affected women (Table S12). Specifically, while *Christensenellaceae* R-7 group, *Holdemanella*, *Prevotella*, *Butyrivibrio*, *Subdoligranulum*, *Faecalibacterium*, and *Senegalimassilia* negatively correlated with women suffering from PCOS, the abundance of *Bacteroides*, *Alistipes*, *Lachnospiraceae* UCG-008, *Escherichia*-*Shigella*, *Parabacteroides*, *Ruminococcus* *gnavus* group, *Faecalitalea*, *Atopobium*, and *Oscillibacter* were significantly higher (Table S12). Therefore, this analysis not only confirmed that PCOS affects the relative abundance of highly abundant bacterial genera typical of the human gut microbiota, with a reduction in the abundance of *Prevotella* and a parallel increase of *Bacteroides* and *Parabacteroides*, but also that this syndrome is associated with a reduced abundance of bacterial genera comprising certain widely recognized health-promoting species, i.e., *Faecalibacterium*, and a concomitant abundance increment of opportunistic pathogens, such as *Escherichia*-*Shigella*. Overall, these results emphasized that PCOS is characterized by a modulation of both vaginal and gut microbiota toward a dysbiotic condition.
